# Factors affecting short-term rental first price: A revenue management model

**DOI:** 10.3389/fpsyg.2022.994910

**Published:** 2022-11-16

**Authors:** Diego de Jaureguizar Cervera, Diana C. Pérez-Bustamante Yábar, Javier de Esteban Curiel

**Affiliations:** Department of Business Economic, Rey Juan Carlos University, Madrid, Spain

**Keywords:** revenue management, short—term rental, dwellings for tourism use pricing, behavioral psychology, exploratory factor analysis, multiple linear regression, technology transfer model, first price

## Abstract

The aim of this paper is to conduct a revenue management study, generating a theoretical model that establishes the relationship between the factors of a Short-Term Rental apartment offered on the Airbnb marketplace or similar and its optimal rental price set when the property is first put on the market, considering not only the characteristics defined in the platform listing but also the sociodemographic characteristics of the area in which the apartment is located. The research process was structured in six phases as case study for the technology transfer model. First, research planning was conducted to estimate the time, cost, and suitability of the research topic. Second, the study design was determined to establish a technology transfer model focusing on the theory of mixed revenue management. Third, data collection about the city of Madrid was extracted from two technological databases, namely *SeeTransparent* based mainly on *Airbnb* (28 internal characteristics of the apartment) and *Deskmind Research* (9 sociodemographic variables of the area in which the apartment is located). Fourth, the data were prepared to create a new descriptive variable of the apartments based on geolocation. Fifth, the analysis of this study was applied to explore the correlation between the price charged per night, the 28 internal characteristics of the apartments, and the 9 sociodemographic variables of their surrounding areas. Sixth, with this integrated database, the information was transformed into multivariate inferential statistics through Exploratory Factor Analysis and Multiple Linear Regression, creating a technology transfer model (big data algorithm) that allows revenue managers to set the price of an apartment based on known information, prior to having a history of market reactions. This research process and model consider some of the factors affecting the psychological behavior of tourism consumers. Practical implications of the findings indicate that the size/capacity of the apartments used for Short-Term rentals largely determines the initial rental price set (72%). The equipment offered by the apartments has a moderate impact (18%), and the sociodemographic characteristics of the surrounding area have a minor influence (11%).

## Introduction

The presence of marketplaces such as *Airbnb*, allowing so-called sharing economies to offer peer-to-peer rentals, has raised this market to a different level in terms of volume and number of listings.

Such marketplaces undoubtedly cover a large portion of the demand for overnight stays when traveling. Since the creation of *Airbnb* in 2008, the number of apartments available to rent for short stays has increased, and indeed this platform has become one of the biggest markets, with a strong presence today. In addition, many other companies have begun to offer this product, connecting landlords with short-term tenants, such as *VRBO, Wimdu, Homeaway*, and other marketplaces, as well as experts in traditional hotel accommodation such as *Booking* and *Expedia*. Even estate agencies and hotel companies such as *RoomMate, SpainSelect, GavirRentals*, and *MyCityHome.es* joined the market, as the sector became more professionalized, and progressively more short-term rental accommodation became available to the market.

The development of Short-Term Rental in the current economy as a model of revenue management for these properties, offering fully furnished real estate assets or apartments ready for immediate occupation, offers a significant area of study, in various fields. In this case, the research presented here also considers the psychological behavior of tourism consumers. For example, some research notes that Short-Term Rentals are putting a financial squeeze on housing for regular tenants and further increasing rent and property prices (Horn and Merante, 2017; Barron et al., 2018; Chen et al., 2019; Ayouba et al., 2020; Garcia-López et al., 2020; Koster et al., 2021a; Tood et al., 2021).

However, according to Levin et al. (2002), Sundararajan (2014), and Einav et al. (2016) Short-Term Rental (STR) home-sharing platforms, such as *Airbnb, HomeAway, VRBO*, and *CouchSurfing*, provide digital peer-to-peer (P2P) marketplace for landlords to rent out empty lodging spaces to third parties, thereby increasing availability and consequently adjusting the price as per the offer-demand rule, alleviating the suggested financial squeeze on housing by having a larger offer but reacting more dynamically to macroeconomic factors. Moreover, according to Brotman (2021), based on the results of their regressions, the motivation of property owners is also increasing, as they even consider building small annexes behind their primary residences to rent out to visitors as Short-Term Rentals.

Other authors state that, although home-sharing platforms provide major efficiencies and create economic benefits in certain aspects (Gárate Alvarez and Pennington-Cross, 2021), they also disrupt the existing property and hospitality industries and create disturbances in the neighborhood in which the lodgings are provided (Espinosa, 2016; Kim et al., 2017). However, such experiences forced the market to develop in terms of requiring more detailed contracts and regulations, imposing penalties for certain behaviors, and charging security deposits that also affect tenants’ willingness to pay.

Undoubtedly, one important concern of landlords who offer their properties through these marketplaces is how to maximize their revenues. Hence, one of the most important issues in property management is ensuring the asset is correctly priced to meet demand at the best price while providing an experience at the destination (Ali et al., 2022) as well as maximizing the owner’s income. To achieve optimal pricing and profitability for the landlord, it is important to determine which factors will affect this valuation, both directly and indirectly.

Many publications dealing with Revenue Management focus on optimizing pricing, based on experience and adjustments according to the market. Other studies compare Short-Term Rentals with other types of rentals. However, it would appear that no publications have yet tackled the issue of how professionals in this sector decide the starting price of a short term property rental. Interviews conducted with several professionals suggest that landlords largely tend to decide this starting price based on intuition. Interestingly, there does not appear to be empirical data available indicating the factors on which these professionals are basing their decision. Landlords do seem to adjust the price as they begin to gather historical information on the market’s reaction; hence the method used during the initial period is largely intuitive and manual, with a significant time investment required.

This paper aims to provide a practical tool for professionals in the real estate sector, on how to decide the most suitable starting price for a residential real estate asset, and which factors affect this decision, reducing the time investment required when adding a new listing to the market. It contributes to the literature by using a technology transfer model to transform the big data gathered, enabling the effects of different internal or external factors on prices to be measured and modeled.

*What are the main applications and uses of understanding the factors affecting the starting price of Short-Term Rentals considering a revenue management model?*

By focusing on this analytical perspective, the research presented here aims to answer the following objectives:

• Identify the main applications of factors such as sociodemographic characteristics and property features that are quantitative indicators of Short—Term Rental apartments vs. Pricing, using a technology transfer model.
• Provide future guidelines to develop this technological model proposed here, to include qualitative indicators and correction factors on the grounds of seasonality and market trends.

To test the problems stated before, this research develops a systematic review of the literature to identify the main contributions made to date within this subject area. The results are analyzed by applying Yin’s (2018) case study as a new technology transfer model with data collection from two technological databases and data analysis with multivariate inferential statistics through Exploratory Factor Analysis and Multiple Linear Regression, creating the technology transfer model (big data algorithm). A discussion and future lines of research in this area are then presented.

Following this introduction, section “Literature review” presents the theoretical framework. Section “Methodology development” sets out the methodology used in the study, and section “Analyze of results (Multivariate analysis)” describes the results, enabling revenue managers to set the price of an apartment on the basis of known information, prior to having a history of market reactions. Section “Discussion” presents the discussion, comparing it with similar studies, and section “Conclusion” states the final conclusions, implications, and limitations.

## Literature review

### Revenue management

Two of the key concepts in revenue management are Littlewood’s rule (Littlewood, 1972) and expected marginal seat revenue (Belobaba, 1987). This notion was mainly created for airlines and considered seat availability, competition, as well as the expiration date of the plane ticket. In fact, American Airlines developed the world’s first revenue management system in 1985 (Lentz et al., 2021), considering revenue management to be an essential instrument in terms of matching supply and demand by segmenting customers, based on their purchase intentions and assigning them in a way that would maximize the firm’s revenues (El Haddad et al., 2008; Ivanov and Zhechev, 2012). Revenue management has been an area of interest in academia for many years (Tse and Poon, 2012), with research on topics such as pricing (Shoemaker, 2003, 2005), price fairness (Kimes and Rohlfs, 2007; Kimes and Wirtz, 2007; Kimes and Taylor, 2010), pricing research (Shy, 2008), and decision framing (Tversky and Kahneman, 1981). It has also benefited strongly not only from marketing management research but more profoundly from operations (Talluri and van Ryzin, 2005), as well as the impact on consumers (Choi and Mattila, 2004; Heo and Lee, 2010).

Revenue Management is widely understood to mean the management of pricing and external factors to maximize the profitability of an asset. Matsuoka (2022) states that Revenue Management aims to maximize financial performance by setting different prices for the same offerings. Cha et al. (2017) understood pricing to be a complex task that requires in-depth understanding. It entails, among other things, monitoring competition day by day, optimizing revenue and setting pricing strategies as per Cullen (2015) and Demirciftci and Belarmino (2022), integrating new technologies, such as artificial intelligence and robots as per Buhalis et al. (2019), using big data, as per Choi et al. (2018), and interpreting the right data in real time (Buhalis and Sinarta, 2019). Revenue management and pricing became particularly important as a tool and strategy in Yeoman’s (2022) consideration of the hotel industry.

Organization support is crucial to maintain a collaborative workplace climate (Li and Srinivasan, 2019) and requires fairly advanced skills (Wang and Brennan, 2014). In this sense, training for managers and appropriate IT infrastructure for streamlined revenue management are essential elements (Selmi and Dornier, 2011). Innovation has emerged as the main driver of change in a business sector that needs to be flexible and resilient (Saura et al., 2022).

Revenue management is increasingly based on marketing, with tailored practices such as personalized pricing or personalized rooms (Tu et al., 2018). The integration between operations and marketing—as well as between strategy and tactics—is key for successful revenue management. Hotels adopt key performance indicators that go beyond room revenues, such as REVPar (Revenue per available room), which accounts for all outlets in the property, or GopPar (gross operating profit per available room), which accounts for expenses and cross selling (Ferguson and Smith, 2014). Ivanov and Zhechev (2012) provide a detailed overview of the specific revenue centers, including room division, F&B, function rooms, and spa and fitness facilities.

There is a vast body of literature on the subject of revenue management, an increasingly trending topic applied to many more markets. Vast numbers of articles such as those mentioned above allow pricing to be adapted based on events, in other words, competition strategy, expiration date, spikes in demand due to football matches, and a large number of different factors to be applied and considered continuously. However, this adaptation comes from a starting point, a defined unique price per unit, and yet it would appear that existing research has yet to tackle initial price-setting based on empirical evidence.

When asking revenue managers, in companies such as *MyCityHome.es*, how they set these starting prices, the most common answer is by experience, benchmarking, and personal impression, stating that they do not have a model to perform this calculation, and results are a little too arbitrary for such an important aspect in property revenue management.

### Big data

Big data refers to the collection of a large amount of data that could be used in the future through the application of technology to learn about behaviors, yield statistics, and establish a decision-making model. The use of mobile applications and other technologies for tourism has seen important changes in the twenty-first century (Saura et al., 2017, 2021).

Elshawi et al. (2018) state that, recently, there have been huge advancements in the scale of data routinely generated and collected through almost all human activity, as well as the ability to exploit modern technologies to process, analyze, and understand such data. The intersection of these trends is known as Big Data Science. According to Ali et al. (2016), “In the modern world we are inundated with data” and “It is estimated that we are generating 2.5 quintillion bytes per day.”

Information is becoming more and more accessible, and so the challenge for big data techniques is to transform this information into useful tools. In this particular case, the aim is to be able to model the data using SPSS and transfer all the knowledge acquired from these data using a simple algorithm or formula, bringing into play the concept of technology transfer.

### Technology transfer model

Also known as transfer of technology (ToT), this is the process of transferring technology from one tech-owning entity to another. Soeder et al. (1990) define it as follows: “Technology transfer is the managed process of conveying a technology from one party to its adoption by another party.”

Technology transfer does not have a universal meaning, according to Kremic (2003), and, in the words of Kutter (1991), it takes place “simply by moving a computer from a laboratory in Boston to a university in Manila” or relocating or exchanging personnel, as per NASA Aeronautics (1993). Others consider international technology transfer complete only when the host economy has absorbed, adapted, and resold the technology (Osman-Gani, 1999).

In order to achieve the goal of the technology transfer study, it is also considered as a process with a Setpoint-Comparator-Feedback loop and post-processing output as per Kremic’s (2003) schema. The set of steps proposed in this paper to establish the Short-Term Rental starting price could be used as a technology transfer model or algorithm.

### Short-term rental

There are three widely accepted definitions for the different types of rentals among professionals in the market. Bearing in mind that there are no precise or documented definitions, they can broadly be classified as follows:

Short-Term Rentals: apartment rentals by the day. Rental of the apartment for a period that can range from one night to several weeks. This type of rental can include Vacation Rentals, Tourism Rentals, Study Rentals, Exhibitions Rentals, and Workday Rentals. In terms of regulations, different regions have, indeed, defined the maximum term and placed certain limitations on the number of days the apartment can be rented. Koster et al. (2021b) summarize some of them: STR-hosts occupy the property for at least 50% of the time (O’Sullivan, 2016). San Francisco imposes a 14% hotel tax (i.e., a Transient Occupancy Tax) and a cap of 90 rental days maximum per year (Fishman, 2015). Amsterdam even imposes a maximum cap of 30 rental days per year as of 2019. In the region of Madrid, however, duration is not yet regulated, and so solutions such as a 5-day minimum stay or a maximum of 90 days have been put forward but rejected in the current regulations. The sample used in this paper recorded an average duration of 3.3219 nights per stay.

Season-Term Rentals: apartments rented by the month. This type of rental can be confused among professionals since it is unclear or at times difficult to differentiate from Short-Term Rentals. Nevertheless, typically, the general consensus within the property sector is that it refers to properties rented for between 4 and 12 months. Some countries such as Spain have restricted this type of rental to 12 months, as longer stays would be subject to the Spanish Urban Leases Act (“*Ley de Arrendamientos Urbanos*”) (Jefatura del Estado, 1994).

Long-Term Rentals: apartments rented by the year. As mentioned previously, although there is no precise definition, there are, nonetheless, legal regulations that establish the duration of this rental period. Since this study was conducted in Madrid, Spain, the Urban Leases Act states that such leases will be no less than 6 months and up to 5 or 7 years, if the landlord is a company, unless otherwise agreed by both parties, for a longer lease only. Season-Term Rentals, on the other hand, will never be longer than 12 months (Jefatura del Estado, 1994).

Taking this into consideration, the research presented in this paper defines Short-Term Rentals to be from 1 day to 4 months, Season-Term Rentals from 4 months up to 12 months, and Long-Term Rentals over 12 months.

#### Short-term rental vs. pricing: Property features

##### Surrounding sociodemographic variables

Different authors, such as Shokoohyar et al. (2020), have found that property location has a significant impact on the rental strategy chosen, underscoring the importance of that well-known refrain “location, location, location” in the property market. Authors such as Benítez-Aurioles (2018) and Gunter and Önder (2018) have focussed previously on geolocation, showing that listing prices are related to distance from the city center, and the response time of the host is negatively correlated with such bookings. Prices are influenced by sociodemographic variables in the surrounding area of the property, and Filippas and Horton (2018) noted that the demand for housing decreases when neighbors see a high turnover of people in their residential area.

As stated previously, this study considers up to 9 sociodemographic variables that may be influencing price.

• Resident population.
• Density of resident population.
• Socioeconomic Index (scale 0–10).
• % of second homes (Over total dwellings).
• No. of active businesses (shops).
• No. of Restaurants/Bars/Cafés.
• No. of Traditional hotels/residences.
• No. of Urban public transport stations.
• No. of Cultural venues (cinemas/theater…).

##### Internal property unit features

Adding to the previous paragraph, Shokoohyar et al. (2020) state that “properties with more bedrooms, closer to the historic attractions, in neighborhoods with lower minority rates and higher nightlife vibe are more likely to have a higher return if they are rented out through Short-Term Rental contract.” Cui et al. (2018) assume that “higher-priced homeowners and higher-priced renters are more likely to live in properties with a greater number of bedrooms, near a major employment center, park, or school, as well as in a suburban location[…] school attendance with higher school quality.”

As stated previously, this study considers up to 28 internal variables that may be influencing price. The pre-study variables established are as follows:

• Bathrooms.
• Bedrooms.
• Beds.
• Capacity.
• Available equipment (% Yes).
• Kitchen.
• Washing Machine.
• Heating / Air Conditioning.
• Child Friendly.
• Dryer.
• Pets Allowed.
• Internet.
• Pool.
• Parking.
• Gym.
• Hot Tub.
• Doorman.
• Suitable For Events.
• Wheelchair Accessible.
• Garden.
• Laptop Friendly.
• Workspace.
• Outdoor Grill.
• Patio or Balcony.
• Restaurant.
• Sauna.
• Spa and Wellness Center.
• Terrace View.

These are also the most relevant variables that major marketplaces like *Airbnb* and *Booking* request when completing their listings. This article sets out to demonstrate the relevance of these variables empirically, in this study.

In light of the above, the theoretical approach applied in this research, mixing sociodemographic and internal variables to determine Short-Term Rental prices (based on Yin’s case study), contributes analytically to the subject of revenue management, seeking to provide complementary information. Other studies have focused on price-setting for Short-Term Rental holiday apartments. The study of Shokoohyar et al. (2020, mentioned earlier) is somewhat similar to the research presented here, showing how sociodemographic and internal variables influence rental prices, but using different methodological techniques such as logistic regression and applied to learn practices to predict the rental strategy with the highest rate of return for a given property. Most publications on Revenue Management, such as those mentioned earlier, focus chiefly on adapting the price of a given asset over time, on comparing calendar events and the competition that offers a similar product. However, the extant research seems to have largely neglected the issue of the optimal starting price. This initial price sets a course that can be adjusted over time. If the level of this initial price is set incorrectly, it could lead to a substantial loss in the potential profitability of the asset, as revenue management corrections are carried out progressively on the basis of historical data.

### Current research: *Airbnb* short-term rental apartments in Madrid

Information was gathered initially from several regions in Spain, but during the research process, the decision was made to concentrate solely on the city of Madrid, for the time being, due to several factors.

First, Madrid is a versatile city within this field of study: it offers Short-Term Rental apartments, as well as Vacation Rentals, Tourist Rentals, Study Rentals, Exhibitions Rentals, Workday Rentals, and so on, all included in the Short-Term Rental definition mentioned earlier.

Second, Madrid was also considered as a destination for the purposes of this article because its tourism demand is largely linear, contrary to other regions in Spain such as Marbella or the Balearic Islands, where demand is more seasonal and focused on summer holidays, or Baqueira or the Sierra Nevada where the peak tourist season is in winter.

Third, very reliable and accessible information has been compiled in Madrid, pertaining not only to sociodemographic and environmental factors but also to the internal features of the properties themselves. Other studies conducted previously in Madrid have also used the Deskmind Research (2022) and SeeTransparent Airbnb (2022) databases, which provide up-to-date information on this destination.

Fourth, the personal-professional background of the authors is more concentrated in Madrid. Indeed, they have working experience at an estate agency *MyCityHome.es*, which mainly focuses on Short-Term rentals and whose headquarters are in Madrid, justifying its selection as a destination.

Moreover, the choice of Airbnb Fast Facts (2022) seems appropriate, as its market share is 57.5%, compared with 36.5% held by *Booking.com* according to MyCityHome (2022). Furthermore, *Airbnb* focuses more on Short-Term apartment Rentals whereas *Booking.com* focuses more on hotel rooms. In addition, according to Zubair and Faiqa (2022), there is a certain level of intended user continuation.

In this context, the study makes three assumptions in this research:

- Assumption 1: the real rental price is taken to be the last price displayed in the Airbnb listing before the listing status changed from “available” to “reserved.”
- Assumption 2: the measurable features of the apartments (quantitative variables, such as beds, bedrooms, and bathrooms) have been taken into account, disregarding the non-measurable features such as users’ reviews, decoration style, or others (qualitative variables).
- Assumption 3: the effects of other variables such as seasonality and competitors have not been taken into consideration, since these variables should be subject to daily correction. The price studied in this paper should be considered a base price and subject to future studies and corrections. Therefore, the data take into consideration an average over the real yearly rental prices.

In light of the above, the final goal of this project is to develop a technology transfer model that establishes a correlation between the different features of a property used for Short-Term Rental in Madrid and the ideal starting price, taking into consideration not only the internal characteristics defined in the *Airbnb* listing but also the sociodemographic dimensions of the area where the apartment is located.

In this regard, two hypotheses have been formulated for statistical testing, based on Shokoohyar et al. (2020):

- Hypothesis 1: Price per night is influenced by the internal features of the apartment, considering that the price of the apartment is affected by the internal features of the property.
- Hypothesis 2: Price per night is influenced by the sociodemographic characteristics of the area, considering that the price of the apartment is affected by the sociodemographic elements of the surrounding environment.

## Methodology development

The data used in this study were obtained from the *SeeTransparent* database for Short-Term Rentals about *Airbnb* and from the *Deskmind Research* database for the sociodemographic variables, to focus on revenue management in apartments for Short-Term Rental as a case study in the city of Madrid. The combination of these two sources represents a form of technology transfer to disseminate knowledge about big data and revenue management issues, such as internal factors (Hypothesis 1) and sociodemographic factors (Hypothesis 2) affecting the pricing of Short-Term Rentals for tourism.

In particular, the design of this technology transfer model is based on Yin’s (2018) as one of the most popular case studies in social sciences and for its theoretical implications. This technology transfer model seems to be an appropriate methodological approach in this research in terms of establishing the relationship between the characteristics of a Short-Term Rental apartment offered through *Airbnb* or a similar marketplace and its optimal rental price, considering not only the characteristics defined in the platform listing but also the sociodemographic characteristics of the area in which the apartment is located. Yin’s (2018) involves six stages that have been applied in this research: planning, design, data collection, preparation, analysis, and sharing of information.

### Plan

Research planning was conducted to estimate the time, cost, and suitability of the research topic. The initial plan was to identify a management model for revenue managers using technology transfer from a combination of databases.

### Design

The study design was determined with a view to establishing a technology transfer model focusing on the theory of mixed revenue management and its practical implications for the marketing management of Short-Term Rental apartments. The application of revenue techniques and technological software boosts the profit management of these real estate assets.

### Prepare

The dataset was mainly taken from *Airbnb*, compiling public information and data provided by the listing managers, data obtained through the big data research company, seeTransparent.com, for a sample of more than 1,048,576 day rentals, along with the internal historical data of *MyCityhome*, among others. The study analyses these technological database sources for apartments booked (with a nightly rate) through *Airbnb* between May 2021 and April 2022 (inclusive) focusing on the area of Madrid and the representative listings for this study. In total, 569 properties were selected (Table 1) with at least one booking in the research period. On average, these properties had been booked for 51% of the established period (187 days). Moreover, the internal features of each property are specified in the *Airbnb* database: booking price, day/month/year, rooms, beds, and equipment.

**TABLE 1:** Sample of *Airbnb* database for this research.

		Frequency
Valid	Without price (May21-Apr22)	481
	Without descriptions	13
	Valid cases	569
	Total	1,063

Source: Own elaboration.

The information provided by the *SeeTransparent* database included the apartment coordinates. These coordinates can be used to geolocate each apartment. Through these coordinates, each apartment was linked to data about its surrounding environment. The *Deskmind Research* database, based on data compiled by the INE (National Institute of Statistics), provides sociodemographic information for each census section and was used here as a primary source to establish the characteristics of the apartment’s surrounding area.

### Prepare (univariate analysis)

The data for each apartment were correlated to the features within a 500-m radius of the apartment location, and the aggregated data from these census sections were linked to each property as a new descriptive variable.

**FIGURE 1:**
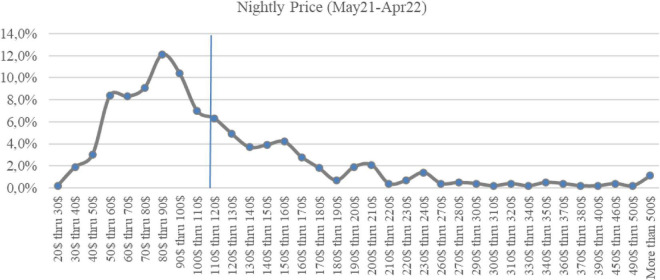
Nightly rate charged (May 2021–Apr 2022) (in $). Source: Own elaboration.

First, the study began with aggregated data collection, working out the nightly rate charged to measure the market-adjusted price based on information from the *SeeTransparent* database and SPSS software (Table 2 and Figure 1).

**TABLE 2:** Average price per night for *Airbnb* properties (May 21–Apr 22) (in $).

*N* (cases)		569
	Mean	Standard deviation
Nightly price (May21–Apr22)	121$	97$

Source: Own elaboration.

Second, the average price per night was calculated for Short-Term Rental apartments booked from May 2021-April 2022. This average was $121 per night with a standard deviation of $97 (Table 2).

Third, the mean values and frequencies of all 28 internal characteristics (number of bathrooms, bedrooms, beds, capacity, kitchen, washing machine…view, terrace) (Table 3). of these 569 Short-Term Rental apartments were calculated. On average, each property has 1.26 bathrooms, 1.39 bedrooms, and 2.08 beds. Furthermore, the vast majority of them have a kitchen (97%), washing machine (90%), heating (89%), and air conditioning (82%).

**TABLE 3:** Between the nightly price with the 28 internal characteristics of the properties.

Equipment available in the properties	Correlation with		
(Description in *Airbnb*)	Nightly price (May 21–Apr 22)		
	N	Pearson’s Corr	Significance
Bathrooms	569	0.420**	0
Bedrooms	569	0.377**	0
Beds	569	0.367**	0
Capacity	569	0.360**	0
Available equipment (% yes)			
Kitchen	569	–0.01	0.819
Washing machine	569	0.066	0.117
Heating	569	0.128**	0.002
Air conditioning	569	0.103*	0.014
Child friendly	569	0.078	0.061
Dryer	569	0.180**	0
Pets allowed	569	0.001	0.981
Internet	569	–0.006	0.894
Pool	569	0.094*	0.025
Parking	569	0.069	0.101
Gym	569	0.079	0.059
Hot Tub	569	0.053	0.206
Doorman	569	0.029	0.497
Suitable for events	569	–0.005	0.897
Wheelchair accessible	569	–0.006	0.893
Garden	569	.c	.
Laptop friendly workspace	569	–0.019	0.653
Outdoor grill	569	.c	.
Patio or balcony	569	.c	.
Restaurant	569	.c	.
Sauna	569	.c	.
Spa and wellness Center	569	.c	.
View	569	.c	.
Terrace	569	.c	.

*Correlation is significant at 0.05 (bilateral). **Correlation is significant at 0.01 (bilateral). c, Not applicable as at least one variable is constant. Source: Own elaboration.

Fourth, the study includes descriptions of the areas surrounding the 569 properties (within a radius of 500 m) taken from the other technological database used (*Deskmind Research*) indicating 9 sociodemographic features of these surrounding locations, such as resident population, the density of resident population, socioeconomic index, % of second homes, number of shops, restaurants, bars, hotels, public transport stations, and cultural venues (Table 4). These factors influence the growth of the market as well as the impossibility of renegotiating due to the common approach taken by governments to defend tenants’ interests. The combination of the different databases allows the collection of multiple data sources and further geolocation analysis.

**TABLE 4:** Description of the area surrounding the 569 properties (500-m radius).

*N* (cases)	569	
Feature	Mean	Standard deviation
Resident population	38,276	12,512
Density of resident population	1,950	638
Socioeconomic Index (scale 0–10)	6.46	1.58
% of second homes (Over total dwellings)	14%	10%
No. of active businesses (shops)	8	9
No. of restaurants/bars/cafés	238	127
No. of traditional hotels/residences	9	12
No. of urban public transport stations	7	5
No. of establishments for cultural shows (cinemas/theaters…)	11	12

Source: Own elaboration.

### Analyze (bivariate analysis)

The analysis developed within this study was applied to explore the correlation between the nightly rate charged and the 28 internal characteristics of the apartments (Table 3). Pearson’s coefficient is between 0.36 and 0.42 for bathrooms, bedrooms, beds, and capacity, indicating a moderate correlation.

The correlation between the nightly rate charged and the 9 sociodemographic features of the surrounding area is shown in Table 5. The highest values for Pearson’s coefficients are found for the number of hotels/residences (0.103), public transport stations (0.099), and cultural venues (0.096). As regards the resident population (-0.084) and population density (-0.084), they present a negative correlation with the nightly rate.

**TABLE 5:** Correlation between the nightly price with the 9 sociodemographic features of the area.

Description of the area surrounding the 569 properties	Correlation with		
(500-m radius) (*Deskmind Research*)	Nightly price (May 21–Apr 22)		
	N	Pearson’s Corr	Significance
Resident population	569	–0.084*	0.045
Density of resident population	569	–0.084*	0.045
Socioeconomic Index (scale 0–10)	569	0.054	0.201
% of second homes (Over total dwellings)	569	0.028	0.512
No. of active businesses (shops)	569	0.041	0.33
No. of Restaurants/Bars/Cafés	569	0.048	0.25
No. of Traditional hotels/residences	569	0.103*	0.014
No. of Urban public transport stations	569	0.099*	0.018
No. of cultural venues (cinemas/theater…)	569	0.096*	0.022

*Correlation is significant at 0.05 (bilateral). Source: Own elaboration.

### Share/transferability (multivariate analysis)

The transferability of the algorithm refers to the degree to which the findings of this case study can be generalized in the context of Short-Term Rental apartments through multivariate inferential statistics such as Exploratory Factor Analysis (EFA) and Statistical Regression Technique (SRT). This technological algorithm can estimate a model in which the market-adjusted price could be established for a Short-Term Rental apartment if the location and internal features are known in advance.

The proposed technological algorithm was first used in an Exploratory Factor Analysis of the main factors (28 internal characteristics and 9 sociodemographic features of the surrounding area) that most influence the market-adjusted price of a Short-Term Rental apartment when the location and internal characteristics are known in advance (37 variables in total, 28 + 9).

## Analyze of results (multivariate analysis)

### Exploratory factor analysis

Exploratory Factor Analysis (EFA) is reduced here to 12 common factors with different descriptive categories and a deep understanding of their influence on the nightly rate. As this subject has never been explored in this way before, EFA was chosen instead of Confirmatory Factor Analysis (CFA) for its validity and reliability. The validity of the new factors was then evaluated using Bartlett’s Test of Sphericity, where *p* < 0.05 indicates that the matrix is adequate due to the high correlations between the variables. Reliability was confirmed using the KMO test (Kaiser–Mayer–Olkin), where a value of at least 0.6 indicates that partial correlations between variables are acceptable.

An Exploratory Factor Analysis was applied to condense these 37 items into a lower number of dimensions. Bartlett’s Test of Sphericity (*p* < 0.000) and the KMO index (0.751 higher than 0.7) justify the application of factor analysis (Table 6).

**TABLE 6:** Bartlett’s Test of Sphericity and the KMO index for this research.

KMO index and Bartlett’s test of sphericity		
Kaiser-Meyer-Olkin measure of sampling adequacy		0.751
Bartlett’s test of sphericity	Approx. chi-squared	6156.398
	Df.	253
	*p*-value	0.000

Source: Own elaboration.

Communalities indicate the amount of variance in each variable that is accounted for. In this EFA, all 38 variables are higher than 0.5, indicating a good explanation capacity for this model (Table 7).

**TABLE 7:** Communalities of this EFA.

Communalities	Initial	Extraction
Density of resident population	1	0.983
Socioeconomic index (scale 0–10)	1	0.947
% of second homes (over total dwellings)	1	0.943
No. of active businesses (shops)	1	0.622
No. of restaurants/bars/cafés	1	0.937
No. of traditional hotels/residences	1	0.949
No. of urban public transport stations	1	0.883
No. of cultural venues (cinemas/theater…)	1	0.951
Bathrooms	1	0.627
Bedrooms	1	0.828
Beds	1	0.778
Capacity	1	0.808
Air conditioning	1	0.801
Dryer	1	0.935
Gym	1	0.793
Heating	1	0.628
Hot tub	1	0.992
Internet	1	0.695
Child friendly	1	0.702
Pool	1	0.777
Washing machine	1	0.759
Doorman	1	0.961
Kitchen	1	0.717
Extraction method: principal component analysis		

Source: Own elaboration.

Several iterations were conducted to reach the optimal number of factors. In particular, the total variance explained by these 12 factors/components is 82.721%, showing that the data are useful (Table 8).

**TABLE 8:** Total variance explained by factor analysis.

Component	Initial eigenvalues			Extraction eigenvalues			Rotation eigenvalues		
	Total	% of variance	% cumulative	Total	% of variance	% cumulative	Total	% of variance	% cumulative
**Total variance explained**									
1	4.17	18.128	18.128	4.17	18.128	18.128	3.863	16.797	16.797
2	3.232	14.054	32.182	3.232	14.054	32.182	2.977	12.945	29.7415
3	2.039	8.866	41.048	2.039	8.866	41.048	1.565	6.803	36.544
4	1.86	8.087	49.135	1.86	8.087	49.135	1.545	6.716	43.261
5	1.393	6.058	55.194	1.393	6.058	55.194	1.517	6.595	49.856
6	1.228	5.34	60.533	1.228	5.34	60.533	1.3	5.651	55.507
7	1.076	4.678	65.211	1.076	4.678	65.211	1.199	5.215	60.722
8	0.95	4.131	69.342	0.95	4.131	69.342	1.036	4.506	65.228
9	0.83	3.607	72.948	0.83	3.607	72.948	1.021	4.44	69.668
10	0.794	3.451	76.399	0.794	3.451	76.399	0.999	4.345	74.014
11	0.77	3.349	79.748	0.77	3.349	79.748	0.999	4.344	78.357
12	0.672	2.923	82.671	0.672	2.923	82.671	0.992	4.314	82.671

Source: Own elaboration.

The rotated component matrix (Table 9) determines factor composition. In this study, 12 factors are identified:

**TABLE 9:** Rotated component matrix and factor labels.

Items involved in the factor	Contribution item- > factor	Factor label
**Factor composition**		
No. of cultural venues (cinemas/theater…)	24%	Surrounding area: Commercial equipment
No. of Traditional hotels/residences	23%	
No. of Urban public transport stations	22%	
No. of Restaurants/Bars/Cafés	20%	
No. of active businesses (shops)	7%	
Density of resident population	78%	Surrounding area: Population density
Socioeconomic Index (scale 0–10)	88%	Surrounding area: Socioeconomic level
% of second homes (over total dwellings)	86%	Surrounding area: Second homes
Bedrooms	27%	Properties for rent: Capacity
Capacity	26%	
Beds	25%	
Bathrooms	19%	
Gym	49%	Properties for rent: Sports equipment (Gym/pool)
Pool	45%	
Child friendly	41%	Properties for rent: Child friendly/internet
Internet	40%	
Washing machine	43%	Properties for rent: Basic appliances (washing machine, kitchen)
Kitchen	43%	
Heating	10%	
Air conditioning	58%	Properties for rent: Air conditioning and heating
Heating	32%	
Dryer	84%	Properties for rent: Complementary appliances: Dryer
Doorman	91%	Properties for rent: Complementary services: Doorman
Hot Tub	97%	Properties for rent: Complementary appliances: Hot tub

Source: Own elaboration.

### Multiple linear regression

Following EFA, Multiple Linear Regression was conducted to define an algorithm (technology transfer model) that explains the average rate charged based on 12 factors of the Short-Term Rental apartments related to the internal property features and the sociodemographic data of the surrounding area.

With an R of 0.754 and an adjusted R squared of 0.569, the model obtained is deemed to be reliable (Table 10).

**TABLE 10:** Summary of this technology transfer model.

Model	R	R-squared	Adjusted R-squared	Estimation standard error
1^b^	0.754^a^	0.569	0.559	32.00081

^a^Predictors: (Constant), Factors 1–12. ^b^Dependent variable: Nightly price (May 21–Apr 22). Source: Own elaboration.

Since the factors are independent of one another, standardized beta coefficients can be used to estimate the weight of each dimension (factor) in terms of the market-adjusted rental price (Table 11).

**TABLE 11:** Coefficients of the technology transfer model.

	Non-standard coefficients		Standard coefficients						
	B	Standard error	Beta	t	Sig.	Beta^ 2^	Factor weight	Main factors affecting the starting prices of this proposed technology transfer model	
**(Constant)**	**110.391**	**1.397**		**79.04**	**0.00**				
Surrounding area: Commercial equipment	11.451	1.404	0.236	8.157	0.00	0.06	**9%**	**Surrounding area**	**11%**
Surrounding area: Population density	–3.347	1.382	–0.07	–2.421	0.02	0.00	**1%**		
Surrounding area: Socioeconomic level	1.969	1.383	0.041	1.424	0.16	0.00	**0%**		
Surrounding area: Second homes	2.734	1.4	0.056	1.952	0.05	0.00	**1%**		
Properties for rent: Capacity	35.595	1.575	0.655	22.599	0.00	0.43	**72%**	**Capacity**	**72%**
Properties for rent: Sports equipment (Gym/pool)	8.815	1.403	0.182	6.283	0.00	0.03	**6%**	**Property equipment**	**18%**
Properties for rent: Child friendly/internet	1.055	1.41	0.022	0.748	0.46	0.00	**0%**		
Properties for rent: Basic appliances (washing machine, kitchen)	1.665	1.422	0.034	–1.171	0.24	0.00	**0%**		
Properties for rent: Air conditioning and heating	8.818	1.374	0.185	6.42	0.00	0.03	**6%**		
Properties for rent: Complementary appliances: Dryer	8.577	1.417	0.175	6.054	0.00	0.03	**5%**		
Properties for rent: Complementary services: Doorman	2.354	1.457	0.047	1.615	0.11	0.00	**0%**		
Properties for rent: Complementary appliances: Hot Tub	2.927	1.387	0.061	2.111	0.04	0.00	**1%**		
a Dependent variable: Nightly price (May 21–Apr 22)					Sum:	0.60	100%		

Source: Own elaboration.

## Discussion

Comparing findings with the existing literature, the research presented here is focused on the geolocation of the property with regard to its sociodemographic surroundings. In this regard, Benítez-Aurioles (2018) and Gunter and Önder (2018) also focus on geolocation, showing that listing price is related to distance from the city center and that the response time of the host is negatively correlated with such bookings.

Additionally, this study considers prices, influenced by the sociodemographic variables of the property’s surrounding area, as positive. In this regard, Filippas and Horton (2018) focus on the decline in the demand for housing if the neighbors fear a high turnover or unfamiliar people in their neighborhood. In contrast, authors such as Yifei et al. (2022) found that location conditions have a limited impact on price in areas with established transportation networks.

Focusing on internal characteristics such as bedrooms, bathrooms, beds, and capacity, in this case, Shokoohyar et al. (2020) argued that “properties with more bedrooms, closer to the historic attractions, in neighborhoods with lower minority rates and higher nightlife vibe are more likely to have a higher return if they are rented out through a Short-Term Rental contract.”

Looking at internal characteristics, Yifei et al. (2022) show that the quality of a property plays a key role in forming the listing prices. The research presented in this paper shows that the nightly rate charged presents a negative correlation between the resident population and population density. In this regard, Espinosa (2016) and Kim et al. (2017) show that Short Term Rental properties also disrupt existing property and hospitality industries and cause disturbance in the surrounding neighborhood.

Other authors, such as Mody et al. (2021) considered the impact on non-hosting residents’ quality of life, finding that they perceived more positive than negative impacts on price. Similarly, the research takes into account data for *Airbnb* in Madrid, identifying the correlation between resident population and population density. In this regard, Martínez-Caldentey et al. (2020) state that tourist rentals arranged through platforms such as *Airbnb* have resulted in over-housing. The historic center of Madrid is an example of this since the *Centro* district is becoming practically a tourist resort, with the largest number of *Airbnb* listings concentrated there.

All the factors studied in this research are factors that may be gathered in a kick off state of the commercialization of the asset and are not dependent on the historical data that may be gathered within time. They are specific to the asset and independent of market competition and calendar events that may also affect the pricing of the apartment.

## Conclusion

This paper aimed to find a behavioral psychology study, generating a revenue management model that establishes the relationship between the factors of a property listed in the *Airbnb* marketplace, or similar, for a Short-Term Rental lease and its optimal rental price set when the property is first put on the market. Findings confirm that the price per night of apartments is influenced by both, the internal features of the property and the sociodemographic characteristics of the area. In this context, some theoretical and practical implications are drawn out in the next lines.

### Theoretical implications

The original theoretical scope of this study was to make a comparison between Short-Term Rental and Long-Term Rental revenue. Nevertheless, during the development of the project, it became clear that this initial goal could not be achieved without establishing an empirical foundation to value the different rental models based on the existing literature and adding to it with these new studies. Therefore, based on Yin’s approach, a technology transfer model for establishing the rental price of Short-Term Rental apartments was chosen as a new theoretical implication, narrowing this down to the internal characteristics of the property and sociodemographic variables of the surrounding area. Revenue managers usually set a starting price for Short-Term Rentals through experience, benchmarking, and personal impression, but they do not have a theoretical model to perform this calculation, and results are a little too arbitrary for such an important aspect of property revenue management.

The statistical relationships arising from Yin’s methodological study and these theoretical developments between price and internal characteristics, and between price and sociodemographic variables, confirm the hypotheses mentioned above. “Hypothesis 1” states that the nightly rate is moderately affected by internal characteristics (refer to Table 3, where Pearson’s coefficient sits between 0.36 and 0.42 for bathrooms, bedrooms, beds, and capacity). “Hypothesis 2” indicates that the nightly rate is slightly influenced by the sociodemographic variables of the property’s surrounding area [refer to Table 5, where the highest values for Pearson’s coefficients are found for the number of hotels/residences (0.103), public transport stations (0.099), and cultural venues (0.096)]. Regarding the resident population (-0.084) and population density (-0.084), this study found a negative correlation with the nightly rate.

With regard to the Exploratory Factor Analysis conducted, 37 variables were reduced to 12 common factors with different descriptive categories and a deep understanding of their influence on the rental price. In particular, the total variance explained by these 12 factors/components was 82.721%, validating the data (refer to Table 8).

Finally, Multiple Linear Regression was applied in order to define an algorithm (technology transfer model) that explains the average rental price based on 12 factors related to the internal characteristics of Short-Term Rental apartments and the sociodemographic features of their surrounding area. With an R of 0.754 and an adjusted R squared of 0.569, the model obtained has a strong predictive/explanatory capacity (refer to Table 10).

### Practical implications

The practical implications of this research involve the implementation of a technology transfer model for revenue management in Madrid based on the main factors affecting the starting prices of Short-Term Rental apartments (refer to Table 11). Essentially, revenue management should be left to pricing decision-makers who are aware of consistent records regarding internal characteristics of the properties and the sociodemographic environment. In particular, the main practical implications of this proposed technology transfer model are:

a) The size/capacity of the properties offered largely determines the Short-Term Rental price (72%).
b) The equipment available in the property influences the Short-Term Rental price to a much lesser extent than its size/capacity (18%).
c) The characteristics of the surrounding area have an even lower impact on the Short-Term Rental price than the previous two factors (11%).

In short, this technology transfer model allows revenue managers and peers to estimate what the market-adjusted price should be for these Short-Term Rental apartments, whose location and internal characteristics are known in advance, as a starting point.

### Limitations and future research

The model chosen achieves different levels of technological development and shows that certain factors significantly affect the price. These factors are quantitative indicators of Short-Term Rental apartments. However, qualitative indicators, such as reviews of the tenant or tourist users have not been included in the model.

Moreover, the data were collected from April 2021 to May 2022 (inclusive). The COVID-19 pandemic may have affected the price, the commitment of the tenant, and their booking process due to travel restrictions and health requirements. Future lines of research will consider correction factors for this technological model on the grounds of seasonality and market trends. Seasonality and market trends will presumably also influence the rental price of Short-Term Rental apartments. Similarly, Long-Term Rental pricing could be studied and compared with Short-Term Rental for these kinds of fully furnished properties.

## Data availability statement

The data collected for this study were extracted from two technological databases, namely *SeeTransparent*, based mainly on *Airbnb* (28 internal characteristics of the apartment), and *Deskmind Research* (9 sociodemographic variables of the surrounding area). Requests to access these datasets can be directed to the corresponding author/s.

## Author contributions

DJ and DP-B conceptualized the theoretical framework, involved in data preparation, and prepared the first draft of the manuscript. DJ and JE performed the data analyses. DJ, DP-B, and JE revised and improved the multivariate analyses, performed and made important contributions linked to the theoretical literature. All authors read and approved the final version of the text.
